# Suicidality in women

**DOI:** 10.1192/j.eurpsy.2021.54

**Published:** 2021-08-13

**Authors:** D. Wasserman

**Affiliations:** National Centre For Suicide Research And Prevention Of Mental Lll-health, Karolinska Institutet, Stockholm, Sweden

**Keywords:** Theories of women's suicidality, Global suicide patterns, Suicide prevention, Suicidal ideation and behaviours

## Abstract

Every year, around 800,000 people die by suicide globally. Whist suicide mortality rates are higher among men, women typically have higher rates of suicidal ideation and behaviours. Despite this fact, suicidality in women is still of grave concern as 71% of women’s violent deaths is accounted for by suicide – a greater percentage than men’s. Suicide patterns among women differ between countries and regions. For example, there is a greater difference in suicide rates between men and women in high-income countries in comparison to low- and middle-income countries. Furthermore, many theories exist to explain women’s suicidality. Yet many of the dominant theories have been challenged from studies in both low- to middle-income and high-income countries. Further research that focuses on the context and culture, rather than the individual, is warranted and will be important for preventative efforts of women’s suicidal behaviours.

**Disclosure:**

No significant relationships.

